# Case Report: The Association Between Chromosomal Anomalies and Cluster A Personality Disorders: The Case of Two Siblings With 16p11.2 Deletion and a Review of the Literature

**DOI:** 10.3389/fpsyt.2021.689359

**Published:** 2021-06-08

**Authors:** Cecilia Maria Esposito, Paolo Enrico, Domenico Sciortino, Elisabetta Caletti, Giulia Bruna Marchetti, Claudia Cesaretti, Lucio Oldani, Alessio Fiorentini, Paolo Brambilla

**Affiliations:** ^1^Department of Pathophysiology and Transplantation, University of Milan, Milan, Italy; ^2^Department of Neurosciences and Mental Health, Fondazione IRCCS Ca' Granda, Ospedale Maggiore Policlinico, Milan, Italy; ^3^Medical Genetics Unit, Woman-Child-Newborn Department, Fondazione IRCCS Ca' Granda, Ospedale Maggiore Policlinico, Milan, Italy

**Keywords:** personality disorder, paranoid personality, genetic, chromosome, duplication, deletion

## Abstract

Although several studies have shown the correlation between chromosomal rearrangements and the risk of developing psychotic disorders, such as schizophrenia, little attention has been given to identifying the genetic basis of pre-disposing personality so far. In this regard, a limited but significant number of studies seem to indicate an association between chromosomal anomalies and cluster A personality disorders (CAPD). Starting from the clinical description of two brothers affected by familial 16p11 deletion syndrome (OMIM #611913), both sharing cluster A and C personality traits, the aim of the present study is to critically review the literature regarding the correlation between chromosomal rearrangements and CAPD. A bibliographic search on PubMed has been conducted, and eight studies were finally included in our review. Most of the studies highlight the presence of schizotypal personality disorder in the 22q11.2 deletion syndrome, whose evolutionary course toward psychotic pictures is well-known. One study also identified a paranoid personality disorder in a patient with a deletion on chromosome 7q21.3. No studies have so far identified the presence of paranoid personality disorder in 16p11 deletion, as in the case of the two siblings we report, while its association with psychosis and autism is already known. Although further epidemiologic studies on broader populations are indicated, our observations might pave the way for the definition of new diagnostic subgroups of CAPD and psychotic disorders, in order to implement the clinical management of such complex conditions.

## Introduction

Genetic studies have provided multiple data on the inheritance of mental disorders, both isolated and within complex syndromes (1). In particular, several chromosomal rearrangements have been associated with a higher risk of schizophrenia (SCZ), autism, or bipolar disorder (BD) (2, 3). Of these, copy number variations (CNVs) involving the 22q11 and 16p11 regions show the highest occurrence of psychotic symptoms, with a documented prevalence of SCZ spectrum disorder of 23.53% in adult patients with the 22q11.2 deletion syndrome (22q11DS) and a 16-fold increased risk of SCZ in individuals with microduplication at proximal 16p11.2 (4–6). The importance of an early identification of such disorders has been demonstrated by the increasing debate about patients at ultra-high risk (UHR) for psychosis over the past years. Thus, it is surprising how little attention has been given so far to the association between prodromal conditions, such as pre-disposing personality disorders, and chromosomal rearrangements (7, 8). In particular, the existence of a cluster A personality disorder (CAPD) may promote the early identification of UHR patients, with the practical implication of a closer monitoring and early management (9–11).

CAPD includes paranoid, schizoid, and schizotypal disorders (PPD, SPD, and STPD, respectively). These different disorders share the tendency to strangeness and eccentricity, resulting in difficult social interactions (12). Beyond environmental and psychosocial risk factors, several studies have shown a significant heritability in CAPD, possibly explained by the neurobiological factors taking part in personality organization (13–15). Following the biopsychosocial pathogenetic paradigm, the evolution of personality disorders generally starts from a genetic pre-disposition, on which environmental triggers and traumatic life events are grafted (16, 17). Thus, the identification of a connection between chromosomal abnormalities and personality disorders may lead to a better understanding of biological mechanisms underlying these psychiatric conditions and the pre-disposing factors to them (18, 19). Moreover, focusing on finding sub-threshold psychotic symptoms often associated with CAPD in patients with a CNV may allow early identification of subjects at risk of developing psychotic disorders in their future (20, 21).

Nowadays, more than 200 syndromes related to recurrent CNVs have been identified, mainly characterized by neurodevelopmental disorders (22), possibly associated with multi-organ involvement. Main symptoms include speech abnormalities, movement disorders, and epilepsy (23, 24). From a psychiatric point of view, patients with chromosomal microdeletion/microduplication syndromes seem to have a higher risk of behavioral alterations and mental disorders than healthy family controls, with a greater risk in males than in females (25). In particular, these rearrangements especially pre-dispose to ADHD (10–38%), anxiety disorders (6–25%), mood disorders (15%), oppositional defiant disorder and other disruptive disorders (13–39%) (26, 27), or autism (up to 1% of autism cases) (28). Furthermore, duplications (but not deletions) have been associated with SCZ in a broad meta-analysis (29). However, the fact remains that psychopathological overlaps seem significant, and the frequency of psychotic symptoms is similar in deletion and duplication carriers (27).

Among the hotspot regions particularly prone to genomic rearrangement is the chromosome numbered 16p11.2: this region, first described in 2009, is sized ~600 kb and contains 27–29 genes (26). A recent review (30) summarizes the clinical aspects of both deletion and duplications occurring in the 16p11.2 region. The presence of psychotic symptoms in patients with 16p11 duplication is ascertained by many authors (4, 26, 27, 31), with a risk of SCZ increased from 8 to 25 times, depending on the studies. As for the deletion, the results are less univocal: according to Niarchou et al. (27), the frequency of psychotic symptoms would essentially be consistent between deletion and duplication carriers, while Giaroli et al. (4) underlined that the association is still controversial. A study by Guha et al. (32) stressed that, while deletions of the proximal portion of 16p11.2 are associated with an increased risk of both SCZ and autism, deletions of the distal portion are more specifically associated with SCZ. On the contrary, other studies described an association between the 16p11.2 microdeletion and autistic traits (25), or found no specific association between 16p11.2 deletion as well as vulnerability and SCZ (29).

Concerning inheritance pattern, both alterations follow an autosomal dominant model with reduced penetrance; most deletions occur *de novo* (60–76%) while most of the duplications are transmitted from an even mildly affected parent (30).

After assisting a patient with an uncommon clinical picture, we have noticed that no case report has so far described the association between 16p11 deletion and personality disorders and that, in literature, only few cases describing the association between chromosomal rearrangements and personality disorders are available so far.

Thus, the aim of our study is to fill such gap, firstly by presenting a literature review on the association between CAPD and chromosomal microduplications or microdeletions and, further, by presenting the cases of two brothers with a familial form of 16p11 deletion syndrome (16p11DS), one of which was affected by PPD.

## Methods

In order to review all scientific papers concerning the correlation between chromosomal microdeletions or microduplications and CAPDs or personality traits, we carried out a comprehensive search on PubMed, with no chronological limits, using the following query: “(^*^delet^*^[title/abstract] OR ^*^duplicat^*^[title/abstract] OR genomic^*^[title/abstract] OR CNV[title/abstract]) AND (Schizotyp^*^[title/abstract] OR Schizoid[title/abstract] OR paranoid[title/abstract] OR ‘cluster A'[title/abstract]) AND (personality[title/abstract] OR trait[title/abstract]) NOT borderline [title/abstract].” The last check was conducted on June 3, 2020.

Headings and abstracts were used to obtain initial search. A manual selection of papers was then performed by two independent operators in order to identify only those concerning the topic of the present article. Each article included was then reviewed in the references to look for any relevant works not found through the query.

Neither design nor time restriction criteria have been established for the choice of articles to be included in this review. Exclusion criteria consisted of (1) paper written in a language different than English; (2) possible co-presence of other known genetic syndromes; (3) off-topic studies; and (4) animal studies. Thus, we included articles in English, concerning the clinical and epidemiological association between chromosomal rearrangements and CAPD.

## Results

Twenty-five papers were initially identified, and 17 were excluded on the basis of the above-mentioned criteria. Finally, eight papers satisfied the inclusion criteria. They are five cross-sectional studies, two cross-sectional and longitudinal studies, and one case report, all conducted between 2001 and 2018 (Table 1). The included articles investigated the association between CAPDs/traits and specific genomic deletions, such as 22q11.2 or 7q21.3 (33–40).

**Table 1:** The association between chromosomal rearrangements and CAPD.

**References**	**Population, (m/f), mean age (years) ± SD**	**Type of genomic anomaly**	**Exclusion criteria**	**Study design**	**Diagnosis, personality and behavioral phenotype**	**Diagnostic/clinical scale**	**Instrumental analysis**	**Study results**
Shapiro et al. (33)	Three groups age and sex matched: SPD *N* = 23, mean age 17,17 ± 2,02 Female 52.17% 22q11DS *N* = 23 Mean age 17.48 ± 2,50 Female 52.17% Healthy controls *N* = 23, mean age 17 ± 2,02 Female 52.17%	22q11.2 deletion	Presence of previously diagnosed mental retardation, current substance abuse or addiction, and any current Axis I diagnosis. Exceptions were the presence of a history of learning disorders as well as attention-deficit and disruptive behavior disorders	Cross-sectional study	Schizotypal personality disorder, 22q11 deletion syndrome	SIPS for all patients; SIDP-IV for SPD patients; WISC-III for participants under the age of 17 WAIS-III for participants 17 and older	FISH for confirming diagnosis in the 22q11DS group	- Both risk groups showed elevated positive, negative, disorganized, and general prodromal symptoms (all *p* < 0.01). - Approximately 60% of individuals in the 22q11DS group and 70% of individuals in the SPD group met symptom criteria for a prodromal psychosis syndrome. - The 22q11DS group scored significantly higher than the SPD group on the “decreased ideational richness” item (*p* < 0.0001) and showed a trend toward greater motor abnormalities (*p* < 0.0001).
Esterberg et al. (34)	SPD: *N* = 30, (19/11), mean age 14.2 ± 1.7 Female 36.7% 22q11DS: *N* = 28, mean age 19.3 ± 4.1 Female 60.7% Healthy controls *N* = 47 Mean age 14.0 ± 1.9 Female 42.6%	22q11.2 deletion	Neurological disorder and any Axis I disorder, including psychotic disorders, autism, and substance use disorders	Cross-sectional study	22q11 deletion syndrome, schizotypal personality disorder (SPD)	SIDP-IV, SCID-I, SIPS, ADI-R	FISH confirming diagnosis in the 22q11DS group	The two groups did not differ on negative, disorganized, or general prodromal symptoms, but were distinguishable based on correlations between prodromal and autistic features (*p* < 0.001); the relationships between childhood autistic features and current prodromal symptoms were stronger for the SPD group (*p* < 0.001).
Ramos-Zaldívar et al. (35)	A 44-year-old man	A 3.191 Mb deletion 7q21.3 (93,389,222–96,579,845) × 1 including DYNC1/1 gene and preserved DLX5/6 gene	/	Case report	7q21.3 deletion without ectrodactyly characterized by craniofacial dysmorphology, paranoid personality disorder, hearing loss, musculoskeletal disorder, inguinal herniae and/or mitral valve prolapse	MMPI-2, scale IQ, House-Tree-Person test	Blood analysis for thyroid function, lipidic profile, and electrolyte levels, and routine biochemical exams. G-band karyotyping FISH with specific probe for Williams-Beuren syndrome SNP array (hg19)	/
Fonseca-Pedrero et al. (36)	22q11DS: *N* = 61 Mean age 14.95 ± 2.13 Healthy controls: *N* = 61 Mean age 15.44 ± 1.76	22q11.2 deletion	History of neurological problems or prematurity; history of any type of psychological treatment or speech therapy; estimated IQ lower than 80; and history of learning difficulties	Cross-sectional and longitudinal study	22q11.2 deletion syndrome, anxiety disorder, ADHD, oppositional defiant disorder, mood disorders, psychotic disorders	SPQ, SIPS, PANSS, BPRS	Not specified	- Adolescents with 22q11DS scored higher than the control group on the interpersonal dimension and suspiciousness subscale of the SPQ (*p* < 0.001). - The analysis of the internal structure of the SPQ in the sample of 22q11DS participants yielded a three-component solution (cognitive–perceptual, interpersonal, and disorganized). Internal consistency coefficients ranged between 0.63 and 0.91. The schizotypal traits were highly stable across a 3.6-year interval and ranged from 0.50 to 0.63. Self-reported schizotypal traits correlated with interview-based ratings of prodromal states and psychotic symptoms
Armando et al. (37)	22q11DS: *N* =111 Mean age 15.7 ± 4.7 Female 53,2%	22q11.2 deletion	Participants with a past or present psychotic disorder	Cross-sectional study	22q11.2 deletion syndrome	UHR symptoms/criteria, SIPS, CGAS or GAF, SCID-I. For participants below 18 years, parents completed DICA-IV. The psychotic disorders supplement of K-SADS-PL. Cognitive assessment: WISC-III, WAIS-III, VIQ, PIQ and FSIQ	Not specified	Contrary to the general population, age only modulated the prevalence of UHR criteria among those with UHR symptoms, but not their prevalence or their clinical significance. This suggests that UHR symptoms might develop as a trait factor in terms of a genetically driven schizotypal disposition in 22q11DS.
Badoud et al. (38)	22q11DS: *N* = 29 Mean age 17.79 ± 2.89 44.8% females Healthy controls: *N* = 27 Mean age 17.25 SD 3.34 44.4% females	22q11.2 deletion	All the potential control participants with a past or present use of psychotropic medication, psychiatric treatment, epilepsy, or any other known neurological condition	Cross-sectional study	22q11.2 deletion syndrome Previous diagnosis of anxiety disorder (12), disruptive disorder (6), major depressive disorder (2)	CBCL or ABCL, WISC-III, WAIS-III, Pictures of Facial Affect by Ekman and Friesen, French computerized adaptation of the Director Task, SPQ, VABS, full-scale IQ	Not specified	- Lower perspective-taking and emotion recognition capacities than typically developing controls. The efficiency of perspective-taking processes (response time) was marginally related to the degree of schizotypal trait expression.
								- the correlation between response time in the perspective condition of the director task and positive schizotypy became marginally significant (p = 0.054). - a positive trend was highlighted between the response times on the perspective taking condition of the director task and levels of negative (*p* = 0.062) and disorganized (*p* = 0.087) schizotypal traits
Schneider et al. (39)	22q11 DS: *N* = 35, (14/21) Mean age 18.06 ± 3.40 60% female Healthy controls *N* = 24, (12/12) Mean age 17.19 ± 2.60 50% female	22q11.2 deletion	History of neurological problems or prematurity, history of any type of psychological treatment or speech therapy, history of learning difficulties, YSR/ASR (Youth Self-Report/Adult Self-Report) total problem score in the clinical range (*t*-score ≥ 64)	Cross-sectional study	22q11 deletion syndrome, schizotypal symptoms	SPQ, Dysfunctional Attitude Scale-Form A (DAS), WISC-III, Verbal Paired Associates Immediate standard score from the Children Memory Scale or the Wechsler Memory Scale, Letter-Number Sequencing standard score from WISC-IV or WAIS-III, Semantic Verbal Fluency Test, BFRT	Quantitative fluorescent polymerase chain reaction (QF-PCR) for confirming 22q11DS	- Adolescents with 22q11DS reported significantly higher score on the negative dimension of the SPQ than controls, even when controlling for the influence of anxiety/depression and intellectual functioning (*p* = 0.005). - Negative and paranoid symptoms were associated with the severity of negative performance beliefs and lower face recognition abilities (*p* < 0.001). - Mediation analyses revealed that negative performance beliefs significantly mediated the association between face recognition and negative/paranoid symptoms (*p* = 0.021).
Armando et al. (40)	22q11DS: *N* = 59, (25/34) Mean age 18.12 ± 4.05 48,84% female Healthy controls *N* = 43 57.63% female	22q11.2 deletion	Patients with intellectual disability (i.e., FSIQ <70)	Cross-sectional and longitudinal study	22q11.2 deletion syndrome, schizotypy, dysfunctional coping	CLES, CERQ, SPQ, SIPS, DICA-IV, Psychotic disorders supplement of the K-SADS-PL, SCID-I, WISC-III, WAIS-III	QF-PCR for confirming 22q11DS. T1-weighted structural MRI, 3-dimensional volumetric pulse sequence, 3-T scanners	−22q11DS group manifests elevated positive, negative, disorganized, and general prodromal symptoms at SIPS, respect to HC; - The 22q11DS group was similar in their SIPS profiles; - Approximately 60% of subjects in the 22q11DS group met criteria for a prodromal psychosis syndrome.

*SPD, schizotypal personality disorder; DS, deletion syndrome; UHR, ultra-high-risk; SPQ, schizotypal personality questionnaire; SIPS, Structured Interview for Prodromal Syndromes; SIDP-IV, Structured Interview for DSM-IV Personality Disorders; WISC-III, Wechsler Intelligence Scale for Children—Third Edition; WAIS-III, Wechsler Adult Intelligence Scale—Third Edition; FISH, Fluorescent in situ Hybridization; SCID-I, Structured Clinical Interview for DSM–IV; ADI-R, Autism Diagnostic Interview-Revised; MMPI-2, Minnesota Multiphasic Personality Inventory-2; SNP, Single nucleotide polymorphism; PANSS, Positive and Negative Syndrome Scale; BPRS, Brief Psychiatric Rating Scale; CGAS, Childhood Global Assessment Scale; GAF, Global Assessment of Functioning; DICA-IV, Diagnostic Interview for Children and Adolescents—Revised; K-SADS-PL, Schedule for Affective Disorders and Schizophrenia for School-Age Children—Present and Lifetime version; VIQ, Verbal IQ; PIQ, performance IQ; FSIQ, full-scale IQ; CBCL, Child Behavior Checklist; ABCL, Adult Behavior Checklist; VABS, Vineland Adaptive Behavior Scales; WISC-IV, Wechsler Intelligence Scale for Children, 4th edition; BFRT, Benton Facial Recognition Test; CLES, Coddington Life Event Scale; CERQ, Cognitive Emotion Regulation Questionnaire; QF-PCR, Quantitative fluorescent polymerase chain reaction*.

### Studies on 22q11.2 Deletion

Most of the included studies concern psychopathological phenotypes associated with 22q11DS (also known as the DiGeorge syndrome). This condition shows different degrees of severity (5) and is variably characterized by congenital heart disease; palate anomalies; facial dysmorphisms; growth retardation; immune deficiencies, especially involving T-cell subset maturation and functioning (due to thymus hypoplasia or aplasia); and increased risk of autoimmune diseases. Vertebral anomalies, risk of hypocalcaemia, and renal or gastrointestinal anomalies are also associated (5). Furthermore, from a neuropsychiatric point of view, in some patients, cortical malformations, epilepsy, tethered cord, and movement disorders are described, and in up to 25% of cases, patients develop SCZ (41, 42).

On the basis of our search, seven studies showed a correlation between 22q11DS and CAPD, with a significantly increased frequency of STPD (with positive, negative, and disorganization symptoms) in comparison to the sporadic variants of the same disorder (33, 34, 36–40). Furthermore, more marked elements of suspiciousness were highlighted in 22q11DS patients with STPD with respect to healthy controls (36, 39), as well as lower perspective-taking and emotion recognition capacities (38). Even when compared to patients diagnosed with STPD without chromosomal abnormalities, patients with 22q11.2 deletion showed a decreased ideational richness and increased motor abnormalities (33).

In several studies, the prodromal role of 22q11.2 deletion has been described, both as an independent risk factor for psychosis and with respect to STPD (33, 34). In particular, the criteria for prodromal psychosis are met in around 60% of patients with 22q11.2 deletion. However, while Shapiro et al. (33) ascribed the increased psychotic risk to the pre-morbid schizotypal personality (36), other authors, such as Esterberg et al. (34), highlighted a specific correlation between the psychotic onset and the autistic behavior, as often observed in 22q11DS patients.

### Studies on Other Genomic Anomalies

Finally, just one study described a case of a CAPD in chromosome rearrangements other than 22q11.2. In particular, the 7q21.3 deletion is a rare chromosomal anomaly variably associated with intellectual disability and split hand–split foot malformation. The case report of Ramos-Zaldívar et al. for the first time shed light on the possible psychiatric involvement in this condition. The authors described a patient with craniofacial dysmorphisms, hearing loss, musculoskeletal disorder, inguinal herniae, and mitral valve prolapse (35). The Minnesota Multiphasic Personality Inventory-2 (MMPI) was performed and highlighted a picture PPD.

## Case Presentation

Our study has been inspired by the observation of the clinical picture of a patient, called Patient A, affected by PPD and hospitalized in the psychiatry ward of Ospedale Maggiore Policlinico of Milan following a hetero-aggressive episode. During the hospitalization, Patient A received a diagnosis of 16p11DS. Thereafter, the patient's sister, who presented physical and behavioral features possibly consistent with a 16p11DS, consented to receiving a genetic evaluation that finally also confirmed in her the diagnostic hypothesis. In this paragraph, we will present the clinical cases of Patient A and his sister (Patient B), with all the clinical, instrumental, and neuropsychological assessments carried out in the two patients. Clinically interesting as it would have been, unfortunately, we have no genetic data regarding the parents, who refused to carry out the array comparative genomic hybridization (CGH).

### Patient A

Patient A is a 36-year-old man who lives at home with his parents and his sister. In the anamnestic interview, his parents reported a eutocic delivery and a normal psychomotor and speech development during his childhood. He was described as a shy and introverted kid, especially since middle school (11–13 years old). He always had few friends, preferring his family's company, but social skills have never frankly appeared dysfunctional. The anamnestic reconstruction did not show repetitive behavior patterns or a narrow area of interest. He had serious scholastic issues, which became evident during middle school and then worsened in high school, where he struggled to achieve the end of the year. After the degree, Patient A starts to work as a cashier in a supermarket, employment that he was still carrying out at the time of his hospitalization. During his early adulthood, Patient A developed a suspicious and argumentative personality, with scarce frustration coping skills and frequent episodes of verbal and physical aggression, mainly directed at his family members. His first aggressive episode against his parents occurred at the age of 19, as a consequence of frustrating events that occurred at school. In 2013, after a further episode of psychomotor agitation, he was hospitalized; he was then discharged with the diagnosis of “unspecified schizophrenia spectrum and other psychotic disorder” and subsequently followed up by the psychiatric service of our department. During the following years, he received several drug therapies, mostly consisting of first-generation antipsychotics (FGAs) and mood stabilizers (MSs). At the time of his last admission, his medications included valproic acid (1,000 mg/day), quetiapine (800 mg/day), and clonazepam (1.5 mg/day).

As for his medical history, Patient A did not refer to past medical or surgical conditions, except for obesity (BMI: 34.5), diagnosed since early childhood. Up to that time, he had never smoked or consumed alcohol or psychoactive substances.

His familial medical history was found to be negative concerning psychiatric disorders, except for his sister, as discussed later.

Patient A came to our attention in September 2019 after a further episode of physical aggression against his parents. He was conducted to the Emergency Unit of Ospedale Maggiore Policlinico of Milan. Here, after a psychiatric evaluation, he voluntarily accepted to be admitted into the inpatient unit of our psychiatric department. In the days prior to the hospitalization, he complained of a growing internal tension and feelings of frustration and rage against his family. Also, paranoid thoughts regarding his family and his co-workers were identified during the psychic examinations. During the first days of hospitalization, he appeared suspicious, vindictive, and socially withdrawn. He kept reporting internal tension and rage. He was treated with oral zuclopenthixol 20 mg/day and delorazepam 2.3 mg/day, which resulted in a progressive improvement of the symptoms. Notably, although he exhibited a suspicious behavior and paranoid thoughts, especially with respect to medical and nursing staff, no full-blown positive psychotic symptoms were evidenced during the psychiatric interviews, without clear persecutory delusions, hallucinations, or disorganized behavior.

In our psychiatric unit, Patient A also underwent neuropsychological testing, in order to assess his global cognitive functioning. The Wechsler Adult Intelligence Scale (WAIS-IV) showed an intelligence quotient (IQ) of 84; the processing speed index was especially low (61). Frontal and executive functioning were assessed using the Trail Making Test (TMT) and the Frontal Assessment Battery (FAB). The TMT score was at lower limits, while the FAB scores were normal, except for the set maintenance subtest, which was deficitary (4). Patient A underwent a psychiatric diagnostic assessment by means of structured clinical interviews. Structured Clinical Interview for DSM-5-Clinical Version (SCID-5-CV) did not evidence any psychiatric disorder. However, Structured Clinical Interview for DSM-5-Personality Disorders (SCID-5-PD) evidenced full PPD and avoidant personality disorder and also traits of SPD and STPD. Further investigation with the MMPI-2 confirmed the presence of the mentioned personality traits and also evidenced significative scores for items regarding depression, anxiety, and low self-esteem. No traits suggestive for a diagnosis of autism were highlighted on a dimensional level (MMPI), confirming the clinical exclusion of the diagnosis.

According to some physical features that Patient A shares with his sister and his parents [such as a minor facial dysmorphism (micrognathia) and the presence of obesity] and an IQ score at the lower end of the normal range of values, a diagnostic suspicion of genetic syndrome was raised. Notably, during his hospitalization, a brain MRI was carried out, in order to exclude an organic cause or anatomical abnormalities that could explain his behavioral disorders. MRI showed a slight bilateral enlargement of the frontal cortical sulci and empty sella. Most of the mentioned findings were described as stable compared to an MRI performed in 2013 (when the patient was 31), except for the new finding of an empty sella. The absence of main brain abnormalities reinforced the suspicion of a genetic cause underlying his clinical picture; thus, a consultation by medical geneticists was carried out, in order to assess the presence of putative genetic conditions. Thus, an array CGH analysis was performed, which showed a microdeletion of ~446 kb in the region p11.2 of chromosome 16.

### Patient B

Patient B is a 34-year-old woman, a full sister of Patient A. After Patient A received the diagnosis of 16p11DS, a genetic assessment was suggested for Patient B too, as she shares some peculiar constitutional traits with her brother, such as obesity. The array CGH analysis confirmed the presence of the same microdeletion in the p11.2 region of chromosome 16, leading to the diagnosis of familial 16p11DS.

At the moment of clinical evaluation, Patient B was 151 cm tall and weighed 126 kg, with an equivalent BMI of 55.2, and she was nullipara. She was born from a eutocic delivery, and the speech and psychomotor development was described as normal. The patient referred to some difficulties in relationships with peers and ascribed to her own shyness and suspiciousness and some bullying issues in her middle and high school years. However, relational skills have always resulted in the norm, and the patient has never shown behavioral alterations attributable to a neurodevelopmental defect. Since primary school, her scholastic record was mediocre, and she left high school when she was 16 to start working initially as a kitchen assistant and then changing to several practical works. Nowadays, she works as a cashier in a supermarket, a job that she has been doing regularly for 8 months. Patient B has no history of psychiatric disorders, with the only exception of an episode of generalized anxiety with insomnia, which occurred in 2019 after her brother's physical aggression and hospitalization in the psychiatric ward. Such symptoms lasted about 1 month and were treated for 10 days with low dosages of oral delorazepam by one of the psychiatrists of our outpatient facility. After this acute episode, which fulfilled the diagnostic criteria for the adjustment disorder with anxiety, Patient B obtained a complete remission of symptoms, without the need for further neuropsychological therapies or follow-up.

As for her medical history, Patient B has been suffering from severe obesity since she was 10 years old, for which she has been followed by the nutrition services, with a hospitalization in the nutrition and metabolic ward in 2013, at the age of 25, and a following outpatient care programme lasting till 2017. During this period, Patient B has been able to lose 70 kg, reducing her BMI from 66 to 55, a value that she is currently maintaining. In 2013, she suffered from insulin resistance, from which she recovered with nutritional care. In the last years, Patient B developed a gonarthrosis, secondary to obesity, yet needing infiltrative treatment. The current drug therapy of Patient B consists of periodic cycles of injective folic acid and iron supplementation. With the exclusion of the above-mentioned brief consumption of delorazepam, Patient B was not prescribed any other psychiatric drug. Patient B has no lifetime history of substance abuse or addiction.

For a better consistency of the case presentation, Patient B has been assessed in our outpatient facilities with the same clinical interviews and neuropsychological tests as her brother.

Patient B's SCID-CV did not highlight the presence of current or past major psychiatric disorders, with the exception of the adjustment disorder with anxiety from which she suffered for 1 month in 2019. Similar to Patient A, the SCID-PD dimensional scoring of Patient B showed sub-clinical avoidant and paranoid personality traits (8 and 5 out of 14, respectively), though not fulfilling the DSM-5 criteria for any full-blown personality disorder. On the other hand, the MMPI-2 did not confirm the same personality profile as SCID-PD; notably, MMPI showed an underreporting profile (L scale *T* score = 77) linked to a deliberate minimization of pathological features in order to give a more positive self-image. Anyway, the MMPI-2 revealed a personality profile featured by conformation to social rules and faith in others, excessive self-confidence, and overestimation of her capacity to fulfill the demands of the surrounding environment. No autistic traits were detected both at a dimensional evaluation with the MMPI and at a clinical psychiatric observation. As for the neuropsychological assessment, the WAIS-IV of Patient B showed an intellectual functioning at the lower level of the norm, with a total IQ score of 86. The attentive and executive functioning assessment was globally in line with the age and the schooling of Patient B. More specifically, the TMT did not reveal any deficit in visual attention and task switching, and the executive functional testing only showed a high score of 5 at the bizarreness index of the Cognitive Estimation Test (CET).

## Discussion

The results of our review support the thesis that chromosomal rearrangements may be related not only to cases of major psychoses but also to CAPD (33–40). The clinical relevance of this evidence lies in the fact that these personality disorders often occur in the prodromal phase of psychotic disorders, constituting the pre-morbid personality profiles with the highest risk of developing psychosis (9–11). Although the current literature focuses on the association between the 22q11.2 deletion, causing the DiGeorge syndrome, and an increased risk of SCZ, nonetheless, other genomic rearrangements have been described in literature. More specifically, these include a case report of a patient presenting CAPD in whom a 7q21.3 deletion has been found, as well as 16p11.2 deletion symptoms.

Furthermore, we describe the case of two siblings with an inherited form of 16p11DS. The two siblings, although with different intensities of psychiatric symptoms, showed paranoid and avoidant traits; in particular, the brother reached criteria above the threshold for a categorial diagnosis of personality disorder, while the sister did not. The greater intensity of these traits, as well as sex, which is a known risk factor for episodes of aggression (43), may explain why the clinical manifestation had a different relevance between the two siblings, bringing only the first to the need for hospitalization in a psychiatry ward. The sister also shows better socio-occupational functioning, requiring only outpatient and occasional psychiatric monitoring, due to the onset of anxious symptoms secondary to life events. From a neuropsychological point of view, both siblings present a borderline IQ score, as shown by the WAIS-IV assessment. This observation seems to be consistent with the phenotypic features so far described by the literature for 16p11DS (25). On the other hand, if the executive functioning assessment demonstrated an impaired performance of Patient A in some domains, with a low score at the set maintenance subtest and a lower normal limit score at the TMT, his sister globally showed scores within the normal limits. Such results reflect the differences in the adaptative functioning between the siblings, consisting in better working and social abilities for Patient B in comparison to Patient A. Other common clinical features of the two subjects included the presence of obesity, which is more severe for Patient B in comparison to Patient A, and minor facial dysmorphisms, such as micrognathia.

If the shared clinical features of the siblings fit with the phenotype described by the updated literature on 16p11DS (23, 24), on the other side, the paranoid and avoidant personality traits represent a novelty, when compared to the current phenotypic description of this syndrome. Based on this, our study indicates the need for a better definition of the 16pq11.2 phenotype, which should explore the correlation not only with psychotic and autistic disorders but also with personality disorders. In fact, unveiling the actual prevalence of 16p11.2 deletion in these psychiatric conditions would contribute to the definition of new diagnostic variants with specific clinical pictures and, possibly, the different pathogenetic mechanisms and the susceptibility to certain therapeutic targets. In this view, our study reports some clinical features, such as low IQ level, obesity, minor facial dysmorphisms, and executive functions deficits, that might motivate further genetic studies in order to exclude the existence of 16p11DS, not only in a patient manifesting psychotic symptoms but also in subjects with paranoid and avoidant personality disorders. Moreover, in light of the brain structural MRI alterations shown by one of our patients, further neuroimaging studies may clarify the role of brain damage in the context of the 16p11DS phenotype. Finally, a deeper knowledge of pathogenetic mechanisms specifically underpinning psychotic symptoms and personality disorders in subjects with 16p11DS and a better understanding of possible implication of the haploinsufficiency of the genes involved in the deletion might give new insights on the pathogenesis of such multifactorial psychiatric disorders.

## Limitations And Conclusions

The first limitation that should be emphasized is the scarcity of studies on the correlation between chromosomal rearrangements and CAPD. In particular, the few studies cited in our review mainly concern 22q11.2 deletion, associated with the DiGeorge syndrome, while the results regarding other chromosomal rearrangements are scarce or null. To our knowledge, 16p11.2 deletion has never been described before in relation to personality disorders, while its association with other neuropsychiatric conditions, especially autism and psychosis, is well-known. A greater number of studies is necessary to establish a definitive correlation between CAPD and both chromosomal rearrangements in general and 16p11.2 deletion in particular. The difficulty of identifying a specific phenotype associated with the 16p11DS motivates the absence of large-scale epidemiological studies and appears extremely limiting in the perspective of a future research. Moreover, even if fascinating, using genetic screening in suspicion of CAPD and facilitating eventual psychotic outbreaks could involve high costs and should be carefully discussed for their possible implication in familial genetic counseling and reproductive risk definition, above all as a consequence of the reduced penetrance of conditions such as 16p11DS.

Therefore, our study aimed at a more specific identification of the clinical phenotype correlated with the 16p11.2 deletion, in the perspective of providing a starting point for studies on a larger scale. In particular, for the first time, our case report highlights the association between the 16p11.2 deletion and not only psychosis but also CAPD. This may allow the identification of a wider spectrum of patients at risk, valuing personality aspects below the threshold of UHR states. In fact, it is known that the presence of CAPD is considered among the prodromal factors that would lead to developing psychosis (7, 8). Recognizing these UHR states early, which would be simpler in cases where a specific chromosomal rearrangement is present, would therefore allow recognition of the psychotic risk before its occurrence and possibly prevent it (9–11).

The identification of specific characteristics that may help a genotype–phenotype correlation would not only lead to a broader knowledge of the clinical picture associated with chromosomal rearrangements but would also pave the way for understanding the pathogenetic mechanisms involved. The definition of a particular clinical subtype associated with 16p11.2 deletion could in fact allow, in the future, a personalized diagnostic approach, with evident advantages from both therapeutic and prognostic points of view.

## Data Availability Statement

The original contributions presented in the study are included in the article/supplementary material, further inquiries can be directed to the corresponding author.

## Ethics Statement

Written informed consent was obtained from the individual(s) for the publication of any potentially identifiable images or data included in this article.

## Author Contributions

CE, PE, and DS wrote the manuscript, with LO help. CC and GM made the genetic evaluation. AF and PB conceived the idea and supervised. All authors contributed to the article and approved the submitted version.

## Conflict of Interest

The authors declare that the research was conducted in the absence of any commercial or financial relationships that could be construed as a potential conflict of interest.
